# Understanding the Impact of Perfluorinated Compounds on Cardiovascular Diseases and Their Risk Factors: A Meta-Analysis Study

**DOI:** 10.3390/ijerph18168345

**Published:** 2021-08-06

**Authors:** Siti Suhana Abdullah Soheimi, Amirah Abdul Rahman, Normala Abd Latip, Effendi Ibrahim, Siti Hamimah Sheikh Abdul Kadir

**Affiliations:** 1 Institute of Pathology, Laboratory and Forensic Medicine (I-PPerForM), Universiti Teknologi MARA, Sungai Buloh 47000, Selangor, Malaysia; ctsuhana85@yahoo.com; 2 Institute of Medical Molecular Biotechnology (IMMB), Faculty of Medicine, Universiti Teknologi MARA, Sungai Buloh 47000, Selangor, Malaysia; 3 Department of Biochemistry and Molecular Medicine, Faculty of Medicine, Universiti Teknologi MARA, Sungai Buloh 47000, Selangor, Malaysia; amirahar@uitm.edu.my; 4 Atta-ur-Rahman Institute for Natural Products Discovery (AuRINS), Faculty of Pharmacy, Universiti Teknologi MARA, Puncak Alam 42300, Selangor, Malaysia; drnormala6351@uitm.edu.my; 5 Department of Physiology, Faculty of Medicine, Universiti Teknologi MARA, Sungai Buloh 47000, Selangor, Malaysia; effendi953@uitm.edu.my

**Keywords:** perfluorinated compound, perfluoroalkyl compound (PFCs), meta-analysis, cardiovascular disease (CVDs), CVDs risk factors

## Abstract

Perfluorinated compounds (PFCs) are non-biodegradable synthetic chemical compounds that are widely used in manufacturing many household products. Many studies have reported the association between PFCs exposure with the risk of developing cardiovascular diseases (CVDs). However, those reports are still debatable, due to their findings. Thus, this review paper aimed to analyse the association of PFCs compound with CVDs and their risk factors in humans by systematic review and meta-analysis. Google Scholar, PubMed and ScienceDirect were searched for PFCs studies on CVDs and their risk from 2009 until present. The association of PFCs exposure with the prevalence of CVDs and their risk factors were assessed by calculating the quality criteria, odds ratios (ORs), and 95% confidence intervals (CIs). CVDs risk factors were divided into serum lipid profile (main risk factor) and other known risk factors. The meta-analysis was then used to derive a combined OR test for heterogeneity in findings between studies. Twenty-nine articles were included. Our meta-analysis indicated that PFCs exposure could be associated with CVDs (Test for overall effect: z = 2.2, *p* = 0.02; Test for heterogeneity: *I*^2^ = 91.6%, CI = 0.92–1.58, *p* < 0.0001) and their risk factors (Test for overall effect: z = 4.03, *p* < 0.0001; Test for heterogeneity: *I*^2^ = 85.8%, CI = 1.00–1.14, *p* < 0.0001). In serum lipids, total cholesterol levels are frequently reported associated with the exposure of PFCs. Among PFCs, perfluorooctanoic acid (PFOA) and perfluorooctane sulfonic acid (PFOS) exposure increased the risk of CVDs than other types of PFCs. Although the risk of PFOA and PFOS were positively associated with CVDs and their risk factors, more observational studies shall be carried out to identify the long-term effects of these contaminants in premature CVDs development in patients.

## 1. Introduction

Perfluorinated compounds are synthetic chemicals produced by 3M companies since the 1940s. PFCs consist of at least one perfluoroalkyl moiety (C_n_F_2n+_1) attached to one or more hydrophilic head groups [1]. PFCs are used in manufacturing products, such as non-sticky kitchenware [2], stain-resistant household products, waterproof clothing, mattresses [3] and food packaging [2]. Other than that, they are used in electronics, automotive, aerospace and firefighting materials [1,3]. PFOA, PFHxS and PFOS are among the most common PFCs used in the industry [3,4,5]. For the past 30 years, PFCs are detected in the environment, such as water, air and soil [6,7,8]. They are also detected in the mother’s breast milk [9], food sources, such as beef and seafood [7,10]. The presence of more than 7 to 11 fluorides made PFCs not biodegradable, due to the stable compound. Defluorination of the structure of the compounds are not possible, since the alkyl chain of PFCs does not have any carbon–hydrogen substitution [11]. The PFCs precursors, such as FTOH, can undergo long-range transport through the atmosphere [12]. It was reported that PFOS is continuous and increasingly found in the ice core sample collected at Devon Nuvanut, Canada, although the large contributor of the source is from Continental Asia [13]. After five decades of production, in 2002, the 3M group had phased out PFOS and some of its derivatives [14]. The European Union (EU) also banned most usage of PFOS and related compounds in 2008 [15]. Unfortunately, many advanced countries, such as China and Korea, are still using these compounds in manufacturing PFCs-based products [3,16,17].

PFOS and PFOA are the most commonly detected PFCs found in the serum, plasma or tissue [18,19,20]. PFCs, such as PFOA, PFHxS and PFOS, were reported not efficiently excreted out from humans through urine and sweat, although their level was detected in the human blood [21,22,23]. These compounds, such as PFOA and PFOS, were found to be accumulated in the human body (8 to 13 ng/mL and 13 to 30 ng/mL, respectively), due to the high biliary reabsorption rate and low levels of excretion in the urine [23,24]. Studies have shown that the main PFOS bioaccumulation target organ is the liver [25,26].

The accumulation of PFOS in the liver has been associated with hepatotoxicity [26]. Besides hepatotoxicity, PFCs can serve as endocrine disruptors, contributing to significant health consequences, such as reproductive toxicity [27], neurotoxicity [28], metabolic dysregulation [29] and cardiovascular toxicity [30]. Recently, many epidemiological studies suggested that PFCs exposure may increase the risk of humans developing diseases, such as cardiovascular diseases (CVDs) [31,32,33]. CVDs are the leading cause of death worldwide [34]. An increase of 14.5% of death was reported from 2006 to 2016, with 17.6 million deaths (95% CI, 17.3–18.1 million) attributed to CVDs [34]. Many countries are concerned about the burden of cardiovascular diseases. In 2011, the United Nations officially documented CVDs as a significant public health issue for non-communicable diseases and urged an ambitious strategy for a drastic disease reduction [35].

Several studies have documented the effects of high concentrations of PFCs on humans [2,16,36], yet a low concentration of PFCs exposure should still be a concern. Recently, low-level PFCs in the environment have been reported to cause gene alteration and may lead to the risk of developing CVDs [37,38]. Cumulated data suggest that the risks of CVDs are raised by environmental exposure [39,40]. The United States Environmental Protection Agency (EPA) issued acceptable levels in drinking water up to 70 ng/L on PFOA and PFOS (individually or combined). However, many states and research scientists claim that EPA guideline is not safe enough [41]. In Canada, drinking water advisory bodies and other organisations have set a limit for various PFCs starting from 10 ng/L to thousands of ng/L [42]. Grandjean and Burdz-Jorgensen [21] had proposed a 1 ng/L approximate level of safe drinking water based on the thresholds of immunotoxicity associated with exposure to PFCs in children at the Faroe Islands.

Most studies investigated the presence of PFCs in patients of CVDs or with CVDs risk factors, such as serum lipid profile, diabetes mellitus, hypertension, atherosclerosis and obesity [4,18,19,20]. Furthermore, those studies were comparing the presence of PFCs in patients with healthy subjects. They reported one or two significant associations with certain PFC, which can be PFOS or/and PFOA. In contrast, there were studies that reported no significant findings between PFCs exposure with CVDs or/and CVDs risk factors. Thus, there is a need to review and analyse these findings to demonstrate which PFCs is statistically significant to be associated with CVDs prevalence. This review aimed to analyse PFCs exposure regardless of their levels of exposure using meta-analysis to demonstrate the association of PFCs exposure in individuals with CVDs or CVDs risk factors.

## 2. Methods

PRISMA guidelines for systematic reviews and meta-analyses [43] were followed. The Comprehensive meta-analysis V3 software (Biostat, New Jersey, USA) was used for the analysis.

### 2.1. Literature Search

The following electronic database was searched for articles that had evaluated the association between PFCs exposure with CVDs and their risk factors between January 2009 and till present with English language restriction: Google Scholar, PubMed and Science Direct. This search used Google Scholar using this strategy: (“explode ‘Perfluorinated compounds, Perfluoroalkyl compounds, Peralkyl substance, PFAS, PFCs’/all subheadings”) and (“explode ‘Cardiovascular disease, CVDs’ text word”) or (“explode ‘atherosclerosis’ text word”) or (“explode ‘metabolic risk’ text word”) or (“explode ‘obesity’ or ‘body weight’ text word”) or (“explode ‘hypercholesterolemia’ text word”) or (“explode ‘hypertension text word”) or (“explode ‘hyperlipidemia’ text word”). A similar strategy was used in searches on PubMed and ScienceDirect. For Google Scholar, ‘Advance Search’ was further applied with; find articles with all the words ‘cardiovascular disease’, with the exact phrase “perfluoroalkyl compounds” and without the word ‘animal’. Abstracts were screened independently by two investigators (SSAS and SHSAK).

### 2.2. Inclusion Criteria

To be eligible, studies had to meet the following conditions:

1. *Publication Type.* Research and review articles were eligible. Conference Proceedings was excluded.
2. *Typesof studies.* Randomised controlled trials (RCTs), cohort and cross-sectional studies were eligible. A narrative review and systematic review were excluded.
3. *Types of participants.* Studies of adults (older than 18 years), adolescents (aged 10–18), children (aged 2–9) and infants. Animal and plant were excluded.
4. *Types of interventions.* Studies that compared PFCs exposure with healthy or non-healthy humans.A selection was made for the PFCs exposure only. No restrictions were made regarding concentrations, duration of exposure, type of CVDs and type of CVDs risks.
5. *Types of outcomes.* Studies were eligible if they assessed either (1) comparing non-diseased and CVDs patients (2) comparing healthy and CVDs risk population (3) comparing healthy and metabolic syndrome population (4) ascertained the prevalence of one or more manifestation of CVDs or CVDs risks diagnosis (5) provided quantitative estimation on the association between PFCs exposure and CVDs/their risk outcomes, including odds ratios (ORs) with 95% confidence intervals (CIs) or mean, standard deviation (SD) or difference in mean and sample size in healthy and non-healthy population.

### 2.3. Data Extraction

Eligible studies were assessed independently by two reviewers using a structured form to abstract information about the objectives, (country and year of publication), study subjects (source, area and age at diagnosis), CVDs/their risk exposures (method of ascertainment and definitions used) and main conclusion. Discrepancies were resolved by discussion or consultation with co-authors (S.H.S.A.K., E.I., N.A.L., A.A.R.).

### 2.4. Data Analysis

The review protocol planned a separate analysis for PFCs exposure for (1) association of PFCs exposure with CVDs, (2) association of PFCs exposure with CVDs risks and (3) association of PFCs exposure with serum lipid. The OR from the highest quartile of PFCs exposure in each PFCs category associate with CVDs (95% CIs) were selected for the rest of the articles, whereas data of mean (ng/mL), standard deviation (SD) and sample size of PFCs concentration in healthy and non-healthy were selected only for Predieri et al.,2015 [44]. As for CVDs risk, the OR from the highest quartile of PFCs exposure in each PFCs category associated with CVDs risk (Metabolic Syndrome, obesity, et cetera) (95% CIs) were selected. Serum lipid profile is one of the parameters employed to assess CVDs risks in an individual. The serum lipid profile consists of several components, such as total cholesterol (TC), triglycerides, high-density lipoprotein (HDL) and low-density lipoprotein (LDL). These components are frequently reported as parameters in assessing CVDs risk or monitoring parameters in CVDs patients. The difference in mean (ng/mL) and sample size between exposed and non-exposed PFCs population with 95% CIs were selected with the significant abnormal concentration in any of serum lipid component (mg/dl) as the outcome for the meta-analysis. As the meta-analysis was conducted from summary figures rather than individual case records, the ORs could not be adjusted for confounders.

#### 2.4.1. Assessment of Overall Effect Size

If at least two studies were available on a specific outcome, meta-analysis were calculated using Comprehensive Meta-Analysis V3. A random-effects model was used to analyse statistical heterogeneity between studies [45]. Meta-analysis was performed to calculate pooled risk estimates and ORs with 95% CIs from eligible studies. (Table 1 and Table 2). Where no ORs were available, mean, standard deviation (SD) or difference in mean and sample size were selected [45]. The magnitude of the test overall effect size was calculated using ORs categories with (1) z = 1.5–2: small; (2) z = 2–3: moderate; (3) z > 3: large effect size [46].

#### 2.4.2. Assessment of Heterogeneity

Heterogeneity was explored using the *I*^2^ statistics, a measure on how much variance between studies can be attributed to differences between studies rather than chance (1) *I*^2^ = 0.30%: No heterogeneity; (2) *I*^2^ = 30–49%: Moderate heterogeneity; (3) *I*^2^ = 50–74%: Substantial heterogeneity; and (4) *I*^2^ = 75–100%: Considerable heterogeneity [72]. A *p* value ≤ 0.10 was regarded to indicate significant heterogeneity [72].

#### 2.4.3. Subgroup Analysis

Subgroup and meta-analysis were performed where PFOS, PFOA and PFHxS were selected base on the most highly studied PFCs by previous researchers [73].

#### 2.4.4. Risk of Bias across Studies

Publication bias was assessed by visual analysis of funnel plots, generated using Comprehensive Meta-Analysis V3, if at least 10 studies were included in a meta-analysis [74]. Roughly symmetrical funnel plots were regarded to indicate low risk, while asymmetrical funnel plots were regarded to indicate a high risk of publication bias [75]. Publication bias was investigated by checking for asymmetry in funnel plots of the logarithm of the study ORs against their standard error. The intercept provides a measure of asymmetry where the larger its deviation from zero, the more pronounced the asymmetry, and based evidence of asymmetry is interpreted on *p* < 0.1 [75]. ‘Trim and Fill’ method was applied to the Funnel Plot [76]. The ‘Trim and Fill’ method estimates potentially missing studies, due to publication bias in the funnel plot and adjusting the overall effect estimate. The fundamental assumption of the ‘Trim and Fill’ method is that the studies with the most extreme effect sizes, either on the left or on the right side, are suppressed. Thus, by adjusting the overall effect estimates with the ‘Trim and Fill’ method [76], a funnel plot based on the bias-corrected overall estimate was derived. Prior to that, the direction of the missing studies with the selection ‘to left of mean’ and ‘random-effect model’ were selected from the software in this review. ‘To left of mean’ was selected as studies in the foregoing illustrative favour the positive direction.

## 3. Results

**Figure 1:**
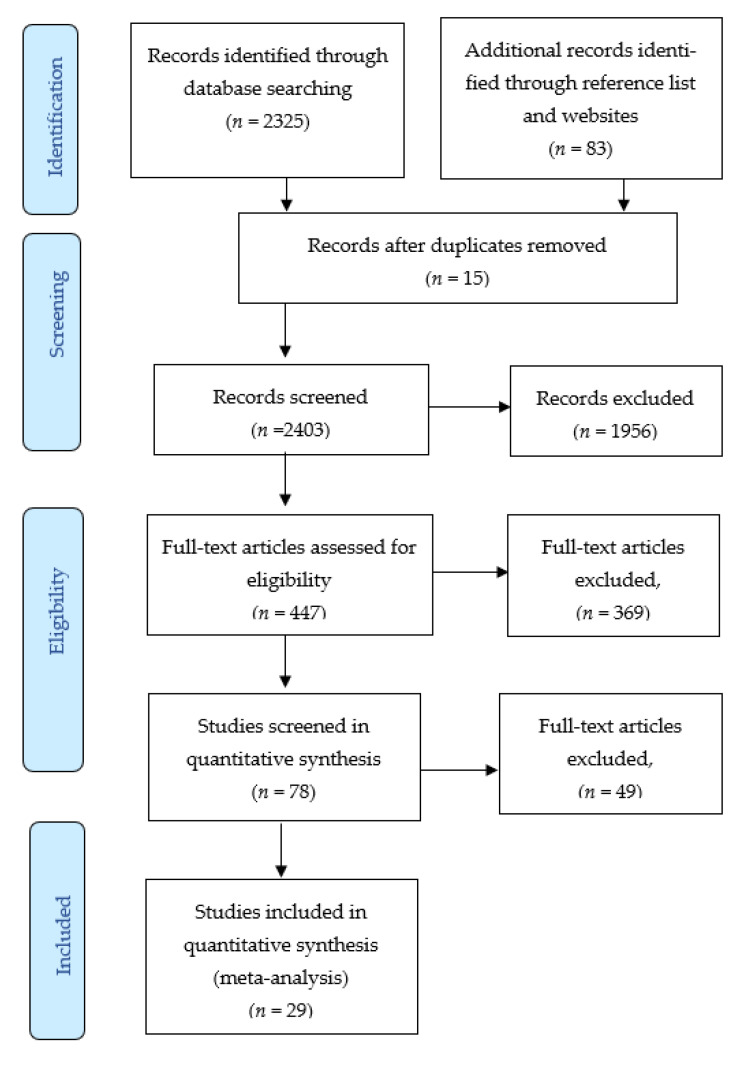
The Flow of Study Identification and Selection.

Figure 1 shows the original database search resulted in 2030 records from Google Scholar, 270 records from Science Direct and 25 records from PubMed. An additional 83 records were identified through the reference list and other websites. After duplication was removed, there were 2403 unique citations eligible for the title and abstract screening. In the first phase of screening, 1956 records in animals were excluded. The second phase of screening excluded 369 articles for the following reason: Advanced search keyword, not in the title of the articles. This left 78 articles assessed for eligibility and screened for quantitative synthesis. The screening excluded 49 articles for the following reason: Thirty were indirectly evaluated the link between PFCs and CVDs, their risk health outcomes and 19 were reporting animal and plant articles. The searches identified fourteen eligible articles using Google Scholar [44,53,54,55,56,57,58,59,60,66,67,69,70,77], a further four through ScienceDirect [61,62,63,64] and another eleven from PubMed [31,33,47,48,49,52,55,65,67,73,78]. A review of reference lists revealed two additional eligible publications [68,71]. Eighteen studies demonstrated the association of PFCs exposure with the prevalence of cardiovascular diseases (CVDs) and their risk factors. Eleven studies have data on serum lipids profile as the outcome [61,62,64,65,66,67,68,69,70,71]. Two studies are excluded from the meta-analysis, since the accessibility of the PFCs exposure is only available to the significant results [73,78]. Each PFCs exposures, and their associations with CVDs and CVDs risk factors, are summarised in Table 1, whereas PFCs exposure associated with serum lipid levels (the main risk factor of CVDs) are summarised in Table 2.

### 3.1. Analysis of Overall Effect and Heterogeneity

**Figure 2:**
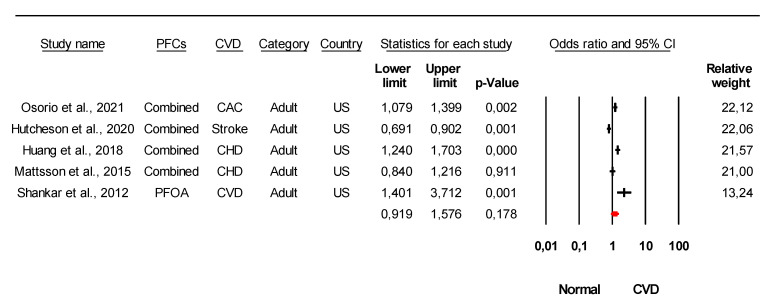
Association of PFCs exposure with CVDs. Meta-analysis using random-effects model, ordered by their date of publication. PFCs combined = combination of more than 2 PFCs. CAC = coronary artery calcium, CHD = coronary heart diseases, CVD = cardiovascular disease. Test for overall effect: z = 2.2, *p* = 0.02; Test for heterogeneity: *I*^2^ = 91.6% Q = 77 df = 4, *p* < 0.0001.

**Figure 3:**
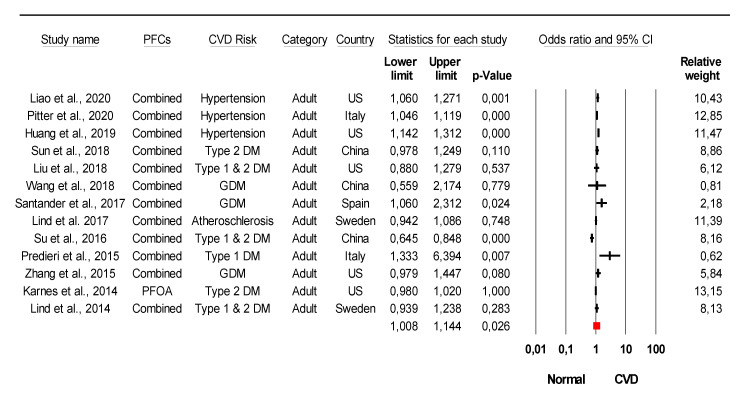
Association of PFCs exposure with their risk factors. Meta-analysis using random-effects model, ordered by date of publication. PFCs combined = combination of more than 2 PFCs. GDM = gestational diabetes mellitus, DM = diabetes mellitus. Test for overall effect: z = 4.03, *p* < 0.0001; Test for heterogeneity: *I*^2^ = 85.8% Q = 84 df = 12, *p* < 0.0001.

**Figure 4:**
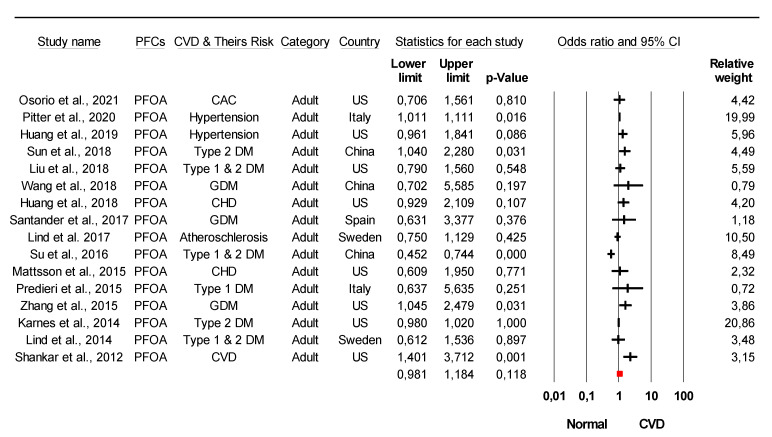
Meta-analysis of studies of PFOA exposure and CVDs and their risk using the random-effects model, ordered by date of publication. Stratified analysis of PFOA as the unit of analysis within the study was selected. CAC = coronary artery calcium, DM = diabetes mellitus, GDM = gestational diabetes mellitus, CHD = coronary heart diseases. Test for overall effect: z = 1.56, *p* = 0.12; Test for heterogeneity: *I*^2^ = 72.1% Q = 53.78 df = 15, *p* < 0.0001.

**Figure 5:**
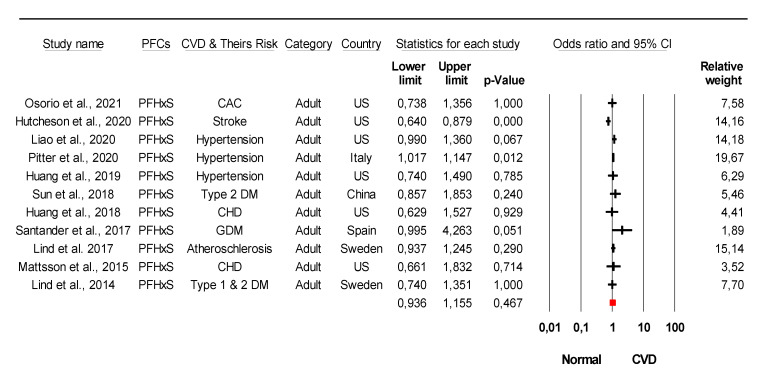
Meta-analysis of studies of PFHxS exposure and CVDs and their risk using the random-effects model, ordered by date of publication. Stratified analysis of PFHxS as the unit of analysis within the study was selected. CAC = coronary artery calcium, DM = diabetes mellitus, GDM = Gestational Diabetes Mellitus, CHD = coronary heart diseases. Test for overall effect: z = 0.73, *p* = 0.47; Test for heterogeneity: *I*^2^ = 58.56% Q = 24.13 df = 10, *p* = 0.007.

**Figure 6:**
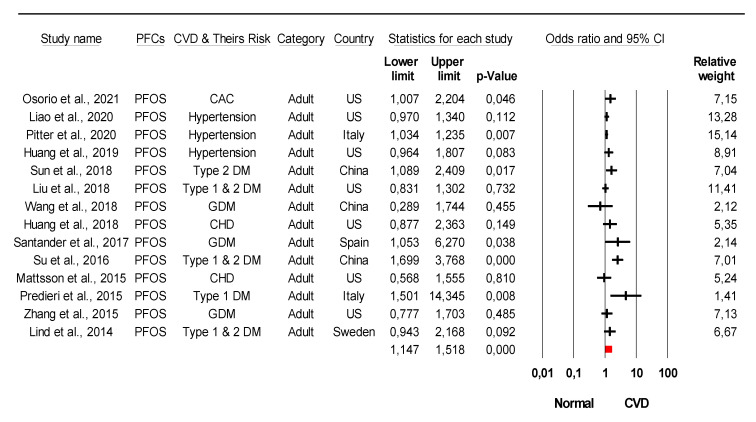
Meta-analysis of studies of PFOS exposure and CVDs and their risk using the random-effects model, ordered by date of publication. Stratified analysis of PFOS as the unit of analysis within the study was selected. CAC = coronary artery calcium, DM = diabetes mellitus, GDM = gestational diabetes mellitus, CHD = coronary heart diseases. Test for overall effect: z = 3.87, *p* < 0.0001; Test for heterogeneity: *I*^2^ = 60.13 Q = 32.66 df = 13, *p* < 0.0001.

**Figure 7:**
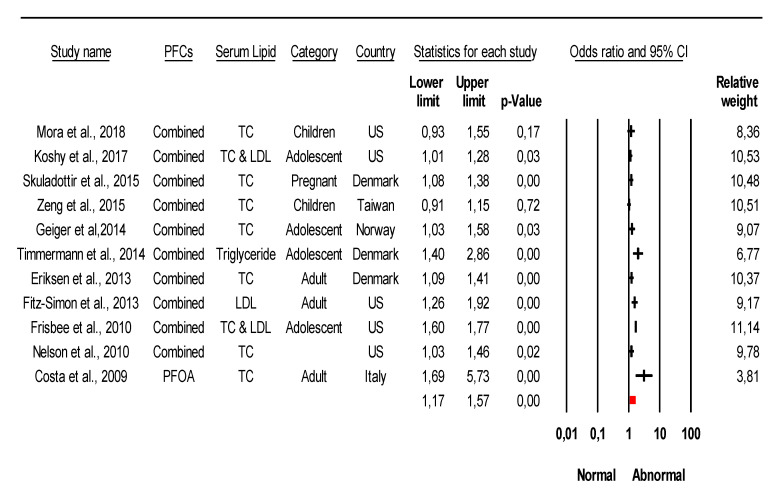
Meta-analysis of studies of PFCs exposure and serum lipid using the random-effects model, ordered by date of publication. TC = total cholesterol, LDL = low density lipoprotein. Test for overall effect: z = 4.04, *p* < 0.0001; Test for heterogeneity: *I*^2^ = 85.2% Q = 110 df = 10, *p* < 0.0001.

The combined meta-analysis results indicated that PFCs exposure might be associated with moderate overall effect on CVDs (z = 2.2, *p* = 0.02) and considerable heterogeneity (*I*^2^ = 91.6% Q = 77 df = 4, *p* < 0.0001) (Figure 2). Strong evidence was observed indicated that PFCs exposure associated with the development of their risk with large overall effect (z = 4.03, *p* < 0.0001) and considerable heterogeneity (*I*^2^ = 85.8% Q = 84 df = 12, *p* < 0.0001) (Figure 3). When stratified analysis done to the subgroup PFOA, meta-analysis indicated that PFOA associated with CVDs and their risk with small overall effect (z = 1.56, *p* = 0.12) and substantial heterogeneity (*I*^2^ = 72.1% Q = 53.78 df = 15, *p* < 0.0001) (Figure 4). No evidence of association identified on PFHxS exposure with CVDs and their risk with overall effect (z = 0.73, *p* = 0.47) and substantial heterogeneity (*I*^2^ = 58.56% Q = 24.13 df = 10, *p* = 0.007) (Figure 5). In contrast, PFOS indicated strong evidence of association with CVDs and their risk with large overall effect (z = 3.87, *p* < 0.0001) and substantial heterogeneity (*I*^2^ = 60.13 Q = 32.66 df = 13, *p* < 0.0001) (Figure 6). Strong evidence was also observed indicated that PFCs exposure associated with serum lipid with large overall effect (z = 4.04, *p* < 0.0001) and considerable heterogeneity (*I*^2^ = 85.2% Q = 235 df = 10, *p* < 0.0001) (Figure 7).

### 3.2. Analysis of Publication Bias

There is evidence of heterogeneity between the studies investigating PFCs exposure with CVDs, serum lipid profiles (CVDs main risk factor) and other risk factors. This is further confirmed by the funnel plots, where a symmetrical funnel shape is obtained (except for meta-analysis on association PFCs exposure with CVDs where the number of studies was less than 10). In the funnel plot, the log ORs represents the natural logarithm of the OR of the individual studies, whereas the standard error represents the standard error in the natural logarithm of the ORs of the individual studies.

**Figure 8:**
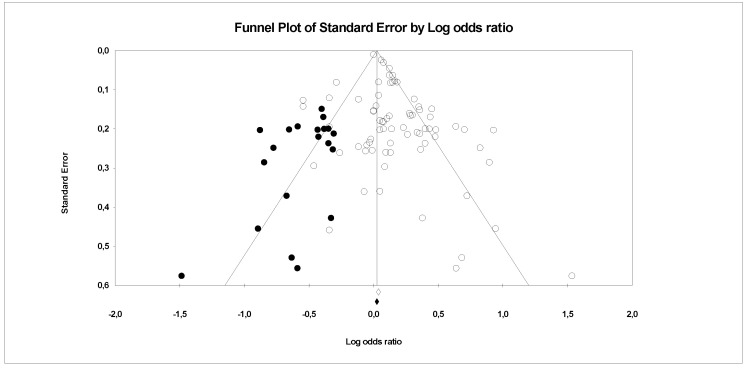
Funnel plots of observational studies on the association of PFCs exposure with their risk factors. The publication bias is adjusted by imputing the missing studies based on the asymmetry of the funnel plot. (●) Plot Imputed and (○) Plot observed studies. Egger’s linear regression test (Intercept 1.4, t = 1, *p* = 0.18). Adjusted values ‘Trim and Fill’ test (1.01, CI = 0.94, 1.08).

**Figure 9:**
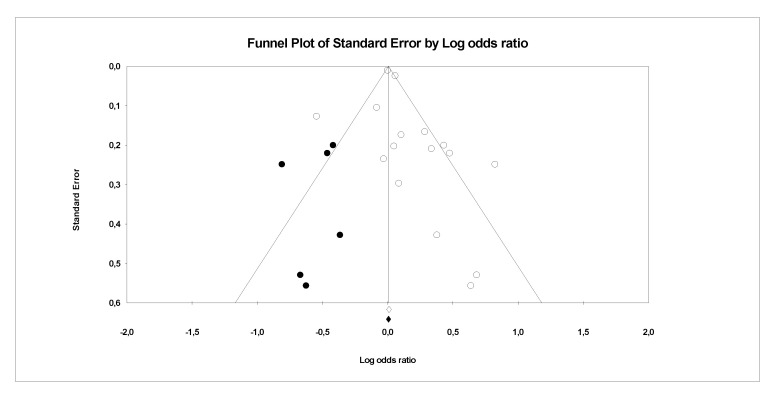
Funnel plots of observational studies of the association of PFOA exposure with CVDs and their risk factors. The publication bias is adjusted by imputing the missing studies based on the asymmetry of the funnel plot. (●) Plot Imputed and (○) Plot observed studies. Egger’s linear regression test (Intercept 0.84, t = 1.66, *p* = 0.12). Adjusted values ‘Trim and Fill’ test (1.00, CI = 0.91, 1.10).

**Figure 10:**
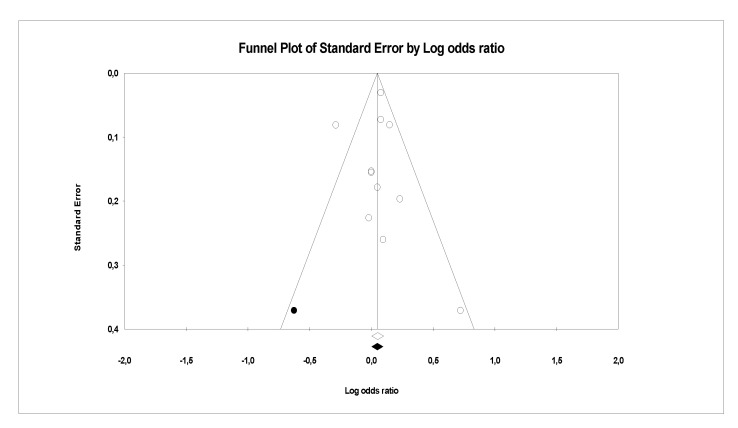
Funnel plots of observational studies of the association of PHxS exposure with CVDs and their risk factors. The publication bias is adjusted by imputing the missing studies based on the asymmetry of the funnel plot. (●) Plot Imputed and (○) Plot observed studies. Egger’s linear regression test (Intercept 0.02, t = 0.03, *p* = 0.10). Adjusted values ‘Trim and Fill’ test 0.98 (0.89, 1.09).

**Figure 11:**
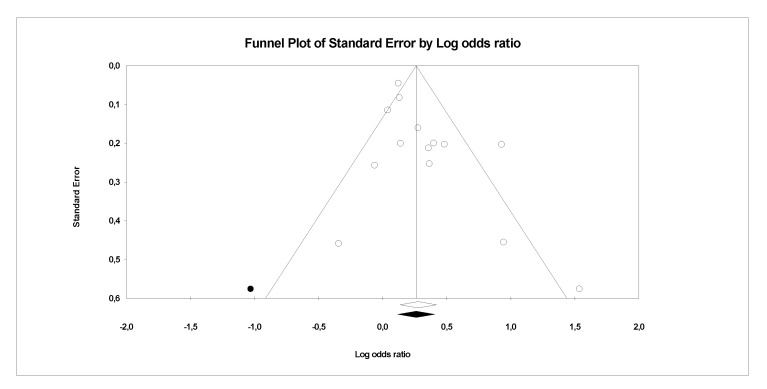
Funnel plots of observational studies of the association of PFOS exposure with CVDs and their risk factors. The publication bias is adjusted by imputing the missing studies based on the asymmetry of the funnel plot. (●) Plot Imputed and (○) Plot observed studies. Egger’s linear regression test (Intercept 1.33, t = 2.3, *p* = 0.04). Adjusted values ‘Trim and Fill’ test (1.30, CI = 1.12, 1.50).

In Egger’s linear regression test, the intercept results indicate that the deviation from zero is not significant for Figure 8 (Intercept 1.4, t = 1, *p* = 0.18), Figure 9 (Intercept 0.84, t = 1.66, *p* = 0.12) and Figure 10 (Intercept 0.02, t = 0.03, *p* = 0.10) suggesting that the plots are symmetry. Whereas significant asymmetry plot is observed in Figure 11 (Intercept 1.33, t = 2.3, *p* = 0.04) and 12 (Intercept −1.93, t = 2.9, *p* = 0.006).

**Figure 12:**
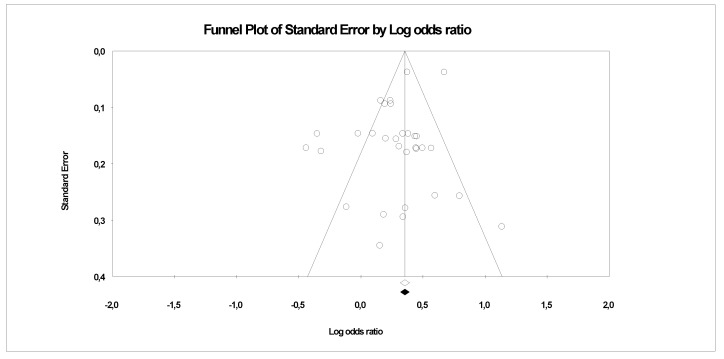
Funnel plots of observational studies of the association of PFCs exposure with serum lipid profile. The publication bias is adjusted by imputing the missing studies based on the asymmetry of the funnel plot. (●) Plot Imputed and (○) Plot observed studies. Egger’s linear regression test (Intercept (−)1.93, t = 2.9, *p* = 0.006).

In ‘trim and fill’ analysis, three studies were trimmed, and 20 possible missing studies (black spot) is indicated in the funnel plot (Association of PFCs exposure with CVDs risk factor, Figure 8). Under the random-effects model, the point estimate and 95% CI for the combined studies is 1.06 (0.99, 1.13). When using ‘trim and fill’, the imputed point estimate is 1.01 (0.94, 1.08). In a stratified analysis of PFOA as the subgroup (Figure 9), six studies are trimmed, and six possible missing studies are indicated. In a stratified analysis of PFHxS as the subgroup (Figure 10), four studies are trimmed and one possible missing study is indicated. Under the random-effects model, the point estimate and 95% CI for the combined studies is 1.04 (0.94, 1.15). When using ‘trim and fill’, the imputed point estimate is 0.98 (0.89, 1.09). Under the random-effects model, the point estimate and 95% CI for the combined studies is 1.08 (0.99, 1.18). When using ‘trim and fill’, the imputed point estimate is 1.00 (0.91, 1.10). In a stratified analysis of PFOS as the subgroup (Figure 11), one study is trimmed, and one possible missing study is indicated. Under the random-effects model, the point estimate and 95% CI for the combined studies is 1.32 (1.15, 1.52). When using ‘trim and fill’, the imputed point estimate is 1.30 (1.12, 1.50). Lastly, in the funnel plot of association of PFCs exposure with serum lipid profile (Figure 12), the ‘trim and fill’ method suggests that no studies are missing. Under the random-effects model, the point estimate and 95% CI for the combined studies is 1.36 (1.17, 1.58). Using ‘trim and fill’, these values are unchanged.

### 3.3. Association of Specific PFC with Cardiometabolic Diseases Based on Epidemiological Studies

Heart failure in diabetic patients can result from myocardial damage. Endothelial dysfunction, inflammation and glycation of atherogenic lipids are significant contributors to heart failure [53,61,79]. However, PFCs association with cardiometabolic diseases are inconsistent among several types of PFCs. PFOS is frequently being reported to be high in individuals with diabetes, followed by PFOA and PFNA. Lin et al., was the first to reveal the correlation of PFCs among adults and adolescents with diabetes using NHANES data [80]. PFCs were also reported to be high in pregnant mothers with gestational diabetes mellitus [54,56,59]. Although inconsistent data reported that either PFOA or PFOS is significantly high, the researchers demonstrated that the concentration of both types of PFCs increased GDM in pregnant mothers when compared to non-pregnant mothers.

Increases in PFNA concentration were found in reduced serum insulin, compromised ß-cell activity and pathological hyperglycemia in adolescents, whereas increased PFOS concentration was found in adults and positively associated with ß-cell activity [80]. Others have been a record of a high concentration of PFOS in children and adolescents with type 1 diabetes mellitus (T1DM), as opposed to control normal subjects [44]. TIDM is an autoimmune disease driven by the activation of T lymphocytes against pancreatic ß-cells, which attacked the pancreatic ß-cells and decreased insulin production [81,82]. Exposure to environmental contaminants can interrupt the production of the immune responses and ß-cell activity, which can potentially increase susceptibility to T1DM [83].

Sufficient data and clear evidence have identified elevated circulating lipid levels as a significant risk factor for the development of atherosclerosis [66,71]. Low-density lipoprotein (LDL) and high-density lipoprotein (HDL) are two types of lipoproteins that play an important role in the transport of fats through the bloodstream [62]. HDL is considered a ‘good’ lipoprotein because it eliminates cholesterol from peripheral tissues back to the liver, and the liver itself eliminates cholesterol. Low HDL and high LDL levels are associated with increased atherosclerosis and coronary artery disorders [84]. LDL can accumulate in the subendothelial space and undergo the chemical modification that further damage the intima when present in excess and may enhance the development of atherosclerotic lesions [85,86,87].

Consistent data in epidemiological studies were observed on the association of PFCs with lipid profiles. Increased in either serum total cholesterol, LDL and triglycerides levels with an increased level of PFCs, especially PFOA and PFOS. 160 medical surveillance on staff working in the PFOA production plant for the past 30 years showed no clinical proof of disease. However, total cholesterol and uric acid increased with serum PFOA levels in a substantial association [71]. Interestingly, another longitudinal study with decline exposure to contaminated drinking water over four years has demonstrated a positive association with a decreased level of LDL [68]. Altered lipid profiles in younger individuals may increase the risk of CVDs [65,69]. Lipid profiles in children [64,66] and adolescents [53,62,65] were also affected when exposed to PFCs in the environment. A high concentration of PFCs was reported in serum office workers suggesting that the office air and dust have a high concentration of PFCs. This may cause by the presence of PFCs in office equipment and carpets [6]. Koshy et al. demonstrated that children who resided and were born between 11 September 1993 to 10 September 2001 and live near the World Trade Centre disaster site have a high concentration of PFCs. The study demonstrated a positive association of increased serum total cholesterol and lipid profile with PFCs exposure [62].

### 3.4. Conflicting Data on the Association of Specific Type PFCs with CVDs from Epidemiological Studies

In the National Health and Nutrition Examination Survey (NHANES) (1996–2000 and 2003–2006), there is an increase in PFOA for CVDs and peripheral arterial disease in a cross-sectional study of 1216 adults. Age, sex, race, smoking history, body mass index, diabetes mellitus, hypertension and serum cholesterol were selected as independent confounders in the study [31]. Another study reported serum levels of 12 major PFCs (PFOA, PFOS, PFHxS, EPAH, MPAH, PFDA, PFBS, PFHP, PFNA, PFSA, PFUA and PFDO) in 10,859 participants from NHANES (1994–2014), but no significant associations, including coronary heart disease and stroke, were observed between PFOA and CVDs [55]. Although the researcher claimed there is no significant association, there are data on the study showing that PFOA and PFHP (p trend; 0.0466 and 0.0472) were significantly associated with stroke when the association was analysed individually. The discrepancy of data interpretation could be due to the reported association of PFOA with total CVDs (congestive heart failure, coronary heart disease, angina pectoris, heart attack and stroke) (*p* for trend = 0.056) and not on specific CVDs [55]. The difference in the sample population may contribute to the conflicting results between studies. Exposure to PFCs was reported to increase up to 10–14% in the population with double income [88]. Individuals of higher education background and income tend to consume more fish, marine food, vegetables and fruits, which are potential sources of PFCs compared to lower socioeconomic status [7,89,90]. Therefore, standardisation of independent confounder selection is important to minimise the inconsistencies of data.

The Carotid Intima-Media Thickness (CIMT) test is used to detect an individual risk of atherosclerotic disorder. The test measures the thickness of the carotid artery inner two layers; the intima and media. A previous cross-sectional study detected four types of PFCs (PFOA, PFOS, PFNA and PFUA ranges from 0.11 to 85.90 ng/L) in random 644 serum samples from Taiwanese adolescents and young adults. Among the four types, PFOS was found to be significantly increased when CIMT increased (*p* < 0.001) [78]. In another study, no significant linear associations were observed between the PFCs and CMIT when the samples of men and women were pooled together [32]. Linear association was statistically plotted to demonstrate a straight-line relationship between these two variables (probability of plaque versus log-transform PFCs concentrations). However, highly important interactions were observed between certain PFCs (PFNA, PFDA and PFUA) and both intimate-media complex and carotid plaque prevalence in women (*p* = 0.002–0.003), whereas these associations were negative in men when analysed base on gender. Both experiments reported a sex-specific role of PFCs in atherosclerosis [77,78]. Testosterone and estradiol were significantly lower in men with a higher level of PFOS in serum. However, a similar pattern of association that linked PFCs to a sex-specific is not established in women [91]. These studies indicated that PFCs may affect men and women differently.

## 4. Discussion

Inconsistent findings were observed in previous studies regarding the association of PFCs exposure with CVDs and their risk factors. Four out of eighteen studies had concluded that PFCs exposure is not associated with CVDs and their risk factors [48,58,92] (Table 1). From the meta-analysis, PFCs exposure to humans might contribute to the CVDs development (Figure 2), with strong evidence indicating the association of PFCs exposure with their risk factors (Figure 3). The risk of stroke among individuals with and without diabetes was not affected by the increase of PFOS and PFHxS exposure [48]. However, other findings concluded that exposure to PFCs significantly increased the risk of getting CVDs [31,32,33,55]. The most recent finding found that prediabetic adults with a higher plasma concentration of PFCs had a higher risk of coronary heart disease and thoracic aorta calcifications [33]. Interestingly, Hutcheson et al. report that the risk of getting stroke is not associated with PFCs exposure, yet the study did not demonstrate whether PFCs increases the risk of stroke, Moreover, the population studied in the report is larger compared to others (50-fold population differences) which might contribute to the insignificant findings.

Although PFOA, PFOS and PFHxS were the most studied PFCs associated with human health, as demonstrated by many previous researchers [73], only PFOA and PFOS exposure were found to be associated with CVDs upon meta-analysis. However, the funnel plot test demonstrated significant asymmetry suggesting publication bias for PFOS (Figure 11). The possible reason is ORs overestimate the relative reduction, or increase, in risk if the event rate is high. This can lead to funnel plot asymmetry if the smaller trials were consistently conducted in patients at higher risk [75]. The asymmetry plot was also observed on the association of PFCs exposure with serum lipid profile. The possible reason might be due to the selection bias on study intervention where the concentration of serum lipid component was selected favours the event of abnormalities (Figure 12).

One previous study demonstrated that almost all PFCs exposure was not associated with CVDs except for PFHP [58]. Although the population selected is based on coronary heart disease adults, yet all subjects were farmers in the rural areas (far from industrial activities). Besides, the study did not declare whether the area or neighbouring area has previously been exposed to army training sites or any industrial activities involving PFCs. Several reports had demonstrated that occupational exposure can be the reason for getting CVDs associated with contaminants [31,50,71].

A recent finding has demonstrated that impaired platelet aggregation, due to PFOA may lead to CVDs risk [79]. The researcher has demonstrated that the platelet membrane is the main site of PFOA accumulation in blood and has shown a massive increase in intra-platelet calcium in PFOA exposed-platelets activated with thrombin receptor peptide 6 (TRAP-6) compared to control (*p* = 0.003). TRAP-6 activates the release of cytosolic calcium of platelets, necessary for the degranulation of platelets and important for the conformation and aggregation of platelets [93]. P-selectin is another platelet activation marker for thrombotic diseases [94]. Human subjects with elevated intimate-media thickness have a high expression of P-selectin, underlying their role in atherosclerosis development [95]. The latest epidemiological findings have found that increases in plasma levels of six PFCs measured were substantially related to changes in carotid intima-medium thickness (increased to 0.058 mm) over the 10-year follow-up period [96]. The in vitro research elucidated the potential triggering effect of PFOA on platelets by examining the expression of P-Selectin in both resting platelets and TRAP-6 active platelets at concentrations ranging from 26 ng/mL to 400 ng/mL after exposure to PFOA [79]. Interestingly, data demonstrated exposure to PFOA provided a significant increase of P-Selectin positive platelets in resting conditions, a similar effect of increased significant P-Selectin positive platelet inactivation with TRAP-6 were also observed [79] This further proven the effect of PFOA in platelet activation.

Meta-analysis is suggesting that there is a need to understand the causative link between PFCs exposure and CVDs at the cellular level. Currently, limited studies are available. A recent finding has demonstrated that microRNAs (miRNAs) such miR-101-3p, miR-144-3p and miR-19a-3p are found to be downregulated when exposed to PFCs in a study involving 239 women drinking water contaminated by firefighting foam. In silico functional analyses suggested that these PFCs-associated miRNAs were annotated to cardiovascular function and disease [97]. Epigenetic changes have appeared as an area to be a further venture in elucidating the association of PFCs with CVDs and their risk factor.

### Limitations

There are several limitations to this study. Firstly, the number of studies included in this work was not large enough to conclude that PFCs exposure is associated development of premature CVDs. Secondly, the number of studies included in this work was not large enough to separate the data into low versus high levels of PFCs exposure or comparing the risk, due to continuous exposure. Thirdly, we are unable to identify PFCs exposure association with serum lipid profile by stratify analysis of both forest plots and funnel plots as the data was not large enough. If more data are available, the analysis will be much clearer and more understandable. Fourthly, T1DM and T2DM were pooled together in previous studies when the researchers analysed the association of PFCs exposure with these CVDs risk factors [53,60]. From our view, understanding the effect of PFCs on diabetes can be improvised by being more specific to T1DM and T2DM before the analysis can be carried out. Since T1DM is the most prevalent chronic metabolic condition in children and adolescents where the ß-cell autoimmune function could potentially trigger by environmental contaminants, such as PFCs [98] Finally, the results might be biased by the inherent limitations of the primary studies, such as confounding factors from unknown or unmeasured parameters. The lack of consistent confounding adjustments may cause overestimation or underestimation of the actual association between PFCs exposure with CVDs and their risk outcomes.

## 5. Conclusions

Our synthesis of adequately designed studies showed a significant association of PFCs exposure with CVDs and their risk factors among children and adults. To our current finding, no available studies on infants for PFCs and CVDs. The heterogeneity between the ORs, indicates true effectiveness between populations. The important highlight in this meta-analysis is, each previous study had concluded a specific type of PFC(s) could be associated with CVDs. However, all the insignificant data of PFCs exposure were included and could not be discriminated during the meta-analyses. This eliminates the possibility of bias in selecting eligible papers that favours CVDs and their risk factors. Our analysis demonstrated that PFOS exposure association with CVDs and their risk are statistically significant. More in vivo and in vitro studies are needed on understanding the mechanism of PFCs exposure effects in the human heart, since several correlations and epidemiological studies do not fully elucidate what exactly happens at the cellular levels. The most recent finding that gives an insight into the cellular event is the effect of PFCs exposure on the platelet aggregation. How PFCs exposure is related to impaired platelet aggregation and leads to CVDs risk can be expanded by understanding the mechanism involving P-selectin and TRAP-6. Several important factors need to be alerted in future studies, such as gender, race and heterogenous lipoprotein subspecies, since these factors were observed to contributes to the inconsistent findings in investigating PFCs exposure association with CVDs and their risk factors.

### The Implication of the Key Findings

Among PFCs, PFOA and PFOS exposure increased the risk of CVDs than other types of PFCs. Although the risk of PFOA and PFOS exposure was positively associated with CVDs and their risk factors, more observational studies shall be carried out to identify the long-term effects of these contaminants with premature CVDs development.

## Figures and Tables

**Table 1:** Summary of PFCs exposure association with CVDs and their risk.

Study	PFCs Association with CVDs and Their Risk	Country	Ref.
**Osorio et al., 2021**	PFOA, PFOS, PFHxS, PFNA, EPAH, MPAH	US	[33]
**Borghese et al., 2020**	PFOA, PFOS, PFHxS	Canada	[47]
**Hutcheson et. al, 2020**	PFOA, PFOS, PFHxS, PFNA	US	[48]
**Liao et al., 2020**	PFOA, PFOS, PFHxS, PFNA	US	[49]
**Pitter et al., 2020**	PFOA, PFOS, PFHxS, PFNA	Italy	[50]
**Huang et al., 2019**	PFOS, PFOA, PFHxS, PFNA, PFHP, PFDE, PFDO, PFBS, PFSA, PFUA	US	[51]
**Sun et al., 2018**	PFOA, PFOS, PFHxS, PFNA, PFUA	China	[52]
**Liu et al., 2018**	PFOA, PFOS	US	[53]
**Wang et al., 2018**	PFOS, PFOA	China	[54]
**Huang et al., 2018**	PFOA, PFOS, PFHxS, PFNA, PFHP, PFDO, PFBS	US	[55]
**Santander et al., 2017**	PFOA, PFOA, PFHxS, PFNA	Spain	[56]
**Lind et al., 2017**	PFOA, PFHxS, PFNA, PFHP, PFSA, PFUA, PFDA	Sweden	[32]
**Su et al., 2016**	PFOA, PFOS, PFNA, PFUA	China	[57]
**Mattsson et al., 2015**	PFOA, PFOS, PFHxS, PFNA, PFHP, PFDO, PFUA, PFDA	US	[58]
**Predieri et al., 2015**	PFOA, PFOS	Italy	[44]
**Zhang et al., 2015**	PFOA, PFOS, PFNA, PFSA,	US	[59]
**Lind et al., 2014**	PFOA, PFOS, PFHxS, PFNA, PFSA, PFUA PFHP	Sweden	[60]
**Shankar et al., 2012**	PFOA	US	[31]

**Table 2:** Summary of PFCs exposure association with serum lipid profile as a CVDs risk factor.

Study	PFCs Associate with CVDs Risk	Serum Lipid	Country	Ref.
**Mora et al., 2018**	PFOA, PFOS, PFHxS, PFDA	TC	US	[61]
**Koshy et al., 2017**	PFOA, PFOS, PFHxS, PFNA, PFDA, PFUA	TC and LDL	US	[62]
**Skuladottir et al., 2015**	PFOA, PFOS	TC	Denmark	[63]
**Zeng et al., 2015**	PFOA, PFOS, PFHxS, PFNA, PFBS, PFHxA, PFHxS, PFDO	TC	Taiwan	[64]
**Geiger et al.,2014**	PFOA, PFOS	TC	Norway	[65]
**Timmermann et al., 2014**	PFOA, PFOS	TG	Denmark	[66]
**Eriksen et al., 2013**	PFOA, PFOS	TC	Denmark	[67]
**Fitz-Simon et al., 2013**	PFOA, PFOS	LDL	US	[68]
**Frisbee et al., 2010**	PFOA, PFOS	TC and LDL	US	[69]
**Nelson et al., 2010**	PFOA, PFOS, PFHxS, PFNA	TC	US	[69,70]
**Costa et al., 2009**	PFOA	TC	Italy	[71]

## Data Availability

The data that support the findings of this study are available from the corresponding author, upon reasonable request.

## Abbreviations

PFOA	Perfluorooctanoic acid
PFOS	Perfluorooctane sulfonic acid
PFDA	Perfluorodecanoic acid
PFDO	Perfluorododecanoic acid
PFHP	Perfluoroheptanoic acid
PFHxA	Perfluorohexanoic acid
PFHxS	Perfluorohexane sulfonic acid
PFNA	Perfluorononanoic acid
PFSA	Perfluorooctane sulfonamide
PFUA	Perfluoroundecanoic acid
PFBS	Perfluorobutane sulfonate
EPAH	2-(N-ethyl-perfluorooctane sulfonamido) acetate
MPAH	2-(N-methyl-perfluorooctane sulfonamido) actetate
FTOH	Fluorotolemer alcohols
